# Loneliness among mothers raising children under the age of 3 years and predictors with special reference to the use of SNS: a community-based cross-sectional study

**DOI:** 10.1186/s12905-018-0625-x

**Published:** 2018-08-16

**Authors:** Marie Mandai, Misato Kaso, Yoshimitsu Takahashi, Takeo Nakayama

**Affiliations:** 1 Center for Genomic Medicine, Kyoto University Graduate School of Medicine, Shogoinkawahara-cho, Sakyo-ku, Kyoto, 606-8507 Japan; 2 Kyoto University School of Public Health, Yoshida Konoe-cho, Sakyo-ku, Kyoto, 606-8501 Japan

**Keywords:** Mothers, Raising children, Loneliness, Social network site (SNS), Social network, Social support, Internal working model, Smartphone

## Abstract

**Background:**

Loneliness in mothers raising children can adversely impact the health of their children and lead to child abuse, depression, and deterioration of mothers’ health. Few studies to date have specifically assessed the association between loneliness and social factors, including the use of social network sites (SNSs), and personal factors. This study aimed to identify predictors of loneliness in mothers raising children, with special reference to SNS use.

**Methods:**

This cross-sectional study involved an anonymous self-reported questionnaire survey of mothers participating in the health check-ups for their children in Nagahama City, Japan, from July 28 to September 29, 2014. The following items were assessed: revised UCLA Loneliness Scale, “Secure” subscale of the Internal Working Model Scale (IWMS-S), psychological distress scale (K6), abbreviated Lubben Social Network Scale (LSNS-6), and types of communication devices and information sources. Multiple regression analysis was performed using the Loneliness Scale score as the dependent variable.

**Results:**

Among 763 mothers attending health check-ups for children in Nagahama City, 715 were available for the survey. Among a total of 638 respondents, data from 523 mothers were analyzed (valid response rate: 73.1%). The mean Loneliness Scale score ± standard deviation was 36.1 ± 9.7. The multiple regression analysis revealed that loneliness was significantly associated with being financially worse-off (β = − 3.35, *p* = 0.004) and struggling (β = − 2.47, *p* = 0.047); having a smaller family social network (β = − 0.32, *p* = 0.032), having fewer friends (β = − 0.49, *p* = 0.001), and having a smaller SNS network (β = − 0.21, *p* = 0.018); a lower secure subscale score on the IWMS-S (β = − 0.56, *p* < 0.001); and a K6 score of ≥5 (β = 4.24, *p* < 0.001).

**Conclusion:**

The degree of loneliness in mothers raising children was associated with a smaller social network, lower secure attachment style, and a higher possibility of psychological distress. These factors should be considered when developing effective interventions against loneliness in mothers raising children.

**Electronic supplementary material:**

The online version of this article (10.1186/s12905-018-0625-x) contains supplementary material, which is available to authorized users.

## Background

Of the various mental health issues that can follow childbirth, a fair amount is known about post-partum depression [1]. However, our understanding of loneliness following childbirth is limited. There is considerable evidence among the elderly that social relationships have a significant impact on health [2–4]. However, whether a similar relationship exists among mothers raising children is unknown. Peplau and Perlman defined loneliness as “the unpleasant experience that occurs when a person’s network of social relations is deficient in some important way, either quantitatively or qualitatively” [5]. Official statistics in Japan have shown that one third of families had a nuclear structure, and 1.6% of families were fatherless [6]. Furthermore, the time spent on childcare by Japanese men with children younger than 6 years is the lowest among many countries [7–9]. In addition to one’s own personal social network, social trust in the neighborhood was reported to be independently associated with the risk of child physical abuse [10]. In general, women with children are considered blessed, likely to be surrounded by friends and family such as their child(ren) and spouse, and are therefore often considered to be immune to loneliness. However, as nuclear families have become the more predominant family structure, and with increasingly weaker regional connections, information pertaining to childrearing is often sparse and difficult to come by, leading to concerns about loneliness in mothers raising children [11]. Mothers who experience a high degree of loneliness are likely to be depressed, which in turn leads to decreased self-esteem and poor health, and consequently the poor health of their children and, in some cases, child abuse [12].

Loneliness is influenced by both personal and social factors [5]. Personal factors include introverted personalities or low self-esteem. Our self-conception is largely based on our relationships with other people [13]. Attachment theory assumes that as individuals construct relationships with others around them, their actions are influenced by whether the other person is useful, as well as by their estimations of whether they are accepted [14]. This theory led to the formation of the Internal Working Model of attachment. Internal Working Models are based on infants’ expectations regarding the accessibility and responsiveness of their caregivers. Hazan and Shaver proposed three patterns of working models in adults that corresponded conceptually to the attachment patterns of children, i.e., “secure,” “anxious/ambivalent,” and “avoidant” [15]. The attachment styles are also personal factors [16].

On the other hand, social factors include social networks and support [17]. Social networks have structural aspects, and social support has subjective traits. Loneliness is likely to be correlated with measures of both social network and social support [4].With the proliferation of Internet use, mothers raising children can now obtain a large amount of information on medical care and child health via the Internet [18]. Social network sites (SNSs) are defined as “web-based services that allow individuals to (1) construct a public or semi-public profile within a bounded system, (2) articulate a list of other users with whom they share a connection, and (3) view and traverse their list of connection may vary from site to site” [19]. SNSs, such as Facebook and Twitter, are web services aimed at members who see value in user-user communication. The current generation of women who experience pregnancy, childbirth, and childrearing uses SNSs on a daily basis [20]. However, no study has examined how SNS use affects loneliness in mothers raising children. It is also unclear how loneliness is associated with personal attachment styles in these mothers. To increase support for this population, it is important to assess the actual level of loneliness experienced by such mothers, and to identify social and personal factors associated with loneliness.

This study aimed to identify predictors of loneliness among mothers raising children in Japan, with special reference to SNS use.

## Methods

### Study participants

This was a cross-sectional study that used the opportunity provided by health check-ups to conduct a questionnaire survey. Health check-ups are held in accordance with the Japanese health care system. Regular collective health check-ups are provided to children during the first four years after birth by each municipal government [21]. A total of 28 group health check-ups for children were held at two local public venues in Nagahama City, Japan, between July and September 2014.

All mothers who attended the health check-ups between July and September 2014 were recruited. Mothers who received questionnaires were registered, and those who completed the questionnaire were considered to have consented to participate in the study.

All participants were mothers raising children under the age of three who resided in Nagahama City at the time of the survey. Participation was voluntary, and all mothers received a verbal and written explanation that they could skip over difficult questions. In order to address any emotional discomfort resulting from filling out the questionnaire, each participant was provided with a tissue package that had the contact information of the researchers and the Nagahama City childrearing consultation center. Women who were unable to answer the Japanese questionnaire (including those who could have answered through a translator) were excluded.

### Questionnaire

The questionnaire was developed based on results of an interview and previous studies on loneliness. The pretest was conducted among women aged 19–39 years, and was followed by revisions. A researcher and local government officials explained the study both in person and in writing to mothers who came for the health check-ups. Mothers who consented to participate were given questionnaires, which were then collected directly from the mother at the site. We used an anonymous self-reported questionnaire.

The questionnaire contained 71 items with the following content: basic characteristics, attachment patterns (patterns showing the tendency towards easy acceptance of help from others), loneliness, psychological distress, social networks (number of associated people and support) [4], and types of communication devices and information sources (see Additional files 1 and 2).

Attachment patterns were evaluated using the Internal Working Model Scale (IWMS) based on the Attachment theory [14, 15, 22]. This model reveals construction patterns of human relationships, which are strongly correlated with loneliness. Interpersonal differences in the Internal Working Model include four patterns that correspond to the attachment patterns observed in infancy/toddlerhood. IWMS comprises three subscales: the “secure scale,” “ambivalent scale,” and the “avoidant scale.” Each subscale has 6 items rated on a 6-point scale. Subscale scores range from 6 to 36 points, with higher scores indicating the distinctive characteristics of attachment patterns. However, we used only the “secure scale” (6 items, score range: 6–36 points), since the concept of “secure” is the most basic of attachment patterns, and to avoid burdening (physically and psychologically) participants in view of the pretest results and previous reports [23]. We used the revised version of the UCLA Loneliness Scale to measure loneliness [24, 25]. This instrument comprises 20 items rated on a 4-point scale. Scores (hereafter, “Loneliness Scale scores”) range from 20 to 80 points, with higher scores indicating a stronger loneliness. Psychological distress was evaluated using the K6 scale [26]. To evaluate social networks, we used the Japanese version of the abbreviated Lubben Social Network Scale (LSNS-6) [4, 27, 28]. For each item on this instrument, the number of associated people in the social network was measured on a 6-point scale. Possible scores range from 0 to 15 points, with higher scores indicating a larger social network. We added “mom friends” and “friends from SNS” as additional options along with “family” and “friend” categories. “Mom friends” were defined as other friends who are mothers acquainted through one’s children.

### Statistical analysis

The primary outcome was loneliness, as evaluated by Loneliness Scale scores. First, we summarized the results from each item of the scale using descriptive statistics. To examine factors associated with Loneliness Scale scores, we calculated mean scores of the revised UCLA Loneliness Scale for each item, and performed either a t-test or analysis of variance (ANOVA). Bonferroni corrections for multiple comparisons were also made to reduce the chance of obtaining Type I errors*.* Any variable deemed significant by univariate analysis, or those found to have a *p* value < 0.2 and were important (either clinically or as reported by previous studies) were identified, and their correlations with Loneliness Scale scores were confirmed using Spearman’s rank correlation coefficient. Following this, the forced entry method was applied in the multiple regression analysis. Loneliness Scale scores were set as the dependent variable, which yielded an estimated partial regression coefficient. The test was two-tailed, and *p* < 0.05 was considered statistically significant. Participants with missing data on the UCLA Loneliness Scale or with missing data for four or more items were excluded. Missing data for each item were not excluded, but treated as one category in univariable analyses. JMP® Pro 11.0.0 was used for statistical analyses.

### Ethical considerations

This study was approved by the Kyoto University Graduate School and Faculty of Medicine Ethics Committee (Approval No. E2248).

## Results

**Fig. 1:**
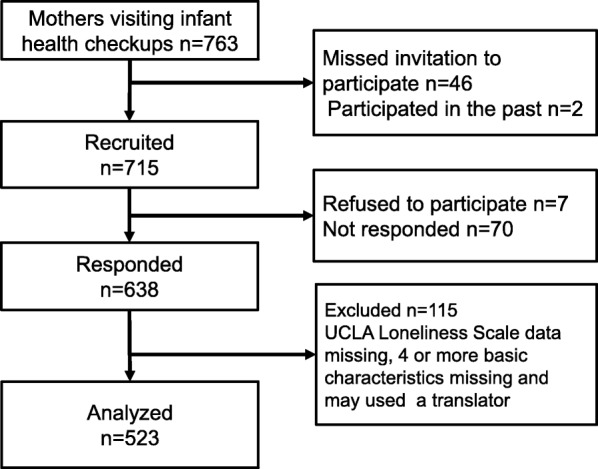
Flowchart of participant selection

Among 763 mothers attending health check-ups for children in Nagahama City, 715 were available for the survey. Two were excluded due to repeated participation, and 46 failed to hand in the questionnaires. A total of 638 mothers returned the questionnaires (89.2%). Of these, those who omitted responses to at least one of the items in the UCLA Loneliness Scale, those with four or more items missing from basic characteristics, or those who may have potentially used a translator were excluded (total of 115 mothers). The remaining 523 questionnaires were subject to analysis (valid response rate: 73.1%) (Fig. 1).

Table 1 summarizes the basic characteristics of participants. Mean age (±SD) was 32.2 (±5.1) years, and 511 participants (98%) were married. Mean Loneliness Scale scores according to each group as categorized by basic characteristics are shown in Table 2. The mean (±SD) Loneliness Scale score for all study participants was 36.1(±9.7) (median, 35; range, 20–74). Even after applying Bonferroni’s correction (*p* < 0.0015), health status (*p* < 0.001) and subjective economic status (*p* < 0.001) were significantly associated with loneliness score. Loneliness Scale scores were highest among teenagers, followed by participants in their 40s and 20s, with the lowest scores observed among those in their 30s. Among married women, those with spouses who did not help with childrearing and housework had higher levels of loneliness. With respect to health status, loneliness tended to increase as health status worsened. For subjective economic status, the lowest loneliness scores were observed in those who reported that they were economically “somewhat stable,” followed by those who reported that they were “stable” and “somewhat unstable.” The highest level of loneliness was reported among those who were economically “unstable.” For education level, graduates of college/graduate school had the lowest Loneliness Scale scores, followed by graduates of trade school/junior college and graduates of junior high, with high school graduates reporting the highest degree of loneliness. Cronbach’s α for the UCLA Loneliness Scale in the present study was 0.77.

**Table 1:** Participant characteristics

	Number	% or mean SD
Age (years), n%		
19 or younger	5	1.0%
20–29	134	25.6%
30–39	308	58.9%
40–49	40	7.6%
No answer	36	6.9%
Mean (SD)		32.2(5.1)
Marital status		
Single	7	1.3%
Married	511	97.7%
Divorced	3	0.6%
No answer	2	0.4%
Help from spouse		
Yes	461	88.1%
No	45	8.6%
No answer	17	3.3%
No. of children		
1	216	41.3%
2 or more	307	58.7%
Employment		
Yes	232	44.4%
No	289	55.3%
No answer	2	0.4%
Daycare		
Attends	217	41.5%
Does not attend	303	57.9%
No answer	3	0.6%
Health status		
Very good	362	69.2%
Good	137	26.2%
Fair	23	4.4%
Poor	0	0.0%
No answer	1	0.2%
Subjective economic status		
Stable	57	10.9%
Somewhat stable	260	49.7%
Somewhat unstable	173	33.1%
Unstable	30	5.7%
No answer	3	0.6%
Live-in parents		
Yes	177	33.8%
No	342	65.4%
No answer	4	0.8%
Education level		
Junior high	27	5.2%
High school	141	27.0%
Vocational/junior college	242	46.3%
University/graduate school	110	21.0%
No answer	3	0.6%
Age of oldest child (months)	43.7(39.0)	

SD, Standard deviation

**Table 2:** Mean (±SD) Loneliness Scale scores

	Loneliness Scale score	*p* value*
Age (years)		0.027
19 or younger	45.0 ± 4.0	
20–29	35.4 ± 9.9	
30–39	35.3 ± 9.3	
40–49	38.6 ± 9.8	
Marital status		0.132
Single	40.0 ± 9.2	
Married	36.0 ± 9.7	
Divorced	44.0 ± 10.1	
Help from spouse		0.002
Yes	35.6 ± 9.5	
No	38.7 ± 10.0	
No. of children		0.948
1	36.1 ± 9.6	
2 or more	36.2 ± 9.7	
Employment		0.520
Yes	35.6 ± 9.3	
No	36.5 ± 9.9	
Daycare		0.115
Attends	36.9 ± 9.8	
Does not attend	35.7 ± 9.5	
Health status		< 0.001
Very good	34.8 ± 9.4	
Good	38.8 ± 9.7	
Fair	41.0 ± 8.8	
Poor		
Subjective economic status		< 0.001
Stable	35.3 ± 9.9	
Somewhat stable	34.8 ± 9.4	
Somewhat unstable	37.2 ± 9.4	
Unstable	43.4 ± 9.0	
Live-in parents		0.769
Yes	36.6 ± 9.5	
No	36.0 ± 9.8	
Highest level of education completed		0.025
Junior high	36.4 ± 9.6	
High school	38.1 ± 9.9	
Vocational/junior college	35.3 ± 9.8	
University/graduate school	35.1 ± 8.8	

*T-test or one-way ANOVA

Data regarding the secure subscale of the IWMS (IWMS-S), abbreviated version of the LSNS-6, and K6, and Spearman’s rank correlation coefficient ρ for Loneliness Scale scores are shown in Table 3. Significant correlations with Loneliness Scale scores were found for the LSNS-6 items of family, friends, mom friends, SNS, IWMS-S, and K6.

**Table 3:** Correlations between loneliness scores and IWMS-S, LSNS-6, and K6

	Number	Percent	Mean	SD	Spearman*	*p* value
Internal Working Model (secure type)	509		20.6	5.5	−0.52	< 0.001
LSNS-6 family	521		8.8	2.7	−0.33	< 0.001
LSNS-6 friends	518		7.1	3.3	−0.43	< 0.001
LSNS-6 mom friends	521		5.0	3.9	−0.32	< 0.001
LSNS-6 SNS	513		6.5	4.8	−0.33	< 0.001
K6	511		3.3	3.5	0.28	< 0.001
-4	363	71.0%				
5–12	139	27.2%				
13-	9	1.8%				

SD, Standard deviation

IWMS, Internal Working Model Scale

LSNS-6, abbreviated version of the Lubben Social Network Scale

*Spearman’s rank correlation coefficient (ρ)

Table 4 shows the results of communication device use and Loneliness Scale scores. All participants used communication devices, of which smartphones were used widely by mothers. Mothers who spent an average of 1–2 h (daily) on the phone within the last 3 months showed the lowest level of loneliness. On the other hand, mothers who spent more than 2 h or fewer than 0.5 h on phones showed higher levels of loneliness. Most mothers did not own a tablet or traditional cell phone. While tablet users tended to show higher levels of loneliness with increased tablet use, no significant correlation was found between Loneliness Scale scores and time spent on personal computers. As the duration of smartphone use became longer, the frequency of SNS use tended to increase (see Additional file 3).

**Table 4:** Communication device use and Loneliness Scale scores

Communication device*	Number	Percent	Loneliness Scale score (mean ± SD)	*p* value
Traditional cell phones	522			0.052
Don’t have	432	82.8%	35.6 ± 9.8	
< 0.5 h	61	11.7%	37.8 ± 8.5	
0.5–1 h	12	2.3%	41.0 ± 8.7	
1–2 h	9	1.7%	43.0 ± 8.4	
2–3 h	6	1.1%	34.8 ± 10.4	
≥ 3 h	2	0.4%	38.0	
Don’t know	0	0.0%		
No answer	1			
Smartphones	522			0.001
Don’t have	65	12.5%	38.4 ± 8.5	
< 0.5 h	48	9.2%	36.4 ± 10.1	
0.5–1 h	113	21.6%	34.9 ± 9.0	
1–2 h	138	26.4%	33.7 ± 9.1	
2–3 h	96	18.4%	37.5 ± 10.1	
≥ 3 h	53	10.2%	38.7 ± 11.3	
Don’t know	9	1.7%	40.8 ± 6.2	
No answer	1			
Tablets	522			0.041
Don’t have	431	82.6%	36.1 ± 9.8	
< 0.5 h	59	11.3%	34.3 ± 8.7	
0.5–1 h	18	3.4%	36.2 ± 9.9	
1–2 h	8	1.5%	39.8 ± 5.8	
2–3 h	5	1.0%	47.8 ± 3.9	
≥ 3 h	1	0.2%	46.0	
Don’t know	0	0.0%		
No answer	1			
Personal computers	521			0.625
Don’t have	268	51.3%	35.8 ± 9.9	
< 0.5 h	190	36.4%	36.1 ± 9.5	
0.5–1 h	26	5.0%	36.7 ± 9.4	
1–2 h	17	3.3%	38.1 ± 9.5	
2–3 h	5	1.0%	42.8 ± 5.3	
≥ 3 h	15	2.9%	36.4 ± 9.2	
Don’t know	0	0.0%		
No answer	2			

*All participants used some type of communication device

Tables 5 and 6 shows Loneliness Scale scores and frequency of consulting with various information sources. Most participants had contacts with “parents”and “friends” as information sources. For these sources, a higher frequency of consultation was correlated with lower levels of loneliness. Relative to those who did not use SNS as an information source, those who did tended to have lower levels of loneliness. A comparison of the various information sources used twice or more per week revealed that Loneliness Scale scores among SNS users were lower than among those who used traditional information sources such as parents, friends, neighbors, specialists and medical personnel (e.g., doctors, midwives, nurses, pharmacists, daycare personnel), television (TV)/radio/newspapers, childrearing seminars, and mothering classes. A higher frequency of consultation with parents and friends (more than once/week) was correlated with a higher frequency of SNS use (see Additional file 4). IWMS-S and frequency of consulting were positively associated with SNS use (see Additional file 5).

**Table 5:** Loneliness and consultation frequency

Sources	Number	Percent	Loneliness Scale score (mean ± SD)	*p* value*
Parents	516			0.018
Never	30	5.8%	38.8 ± 10.6	
Once a year	14	2.7%	41.9 ± 5.7	
Four times a year	45	8.7%	35.2 ± 10.1	
Once a month	138	26.7%	37.3 ± 9.0	
Once a week	123	23.8%	35.1 ± 10.1	
2+ times/week	166	32.2%	35.0 ± 9.7	
Friends	516		<.0001	
Never	56	10.9%	42.1 ± 9.5	
Once a year	39	7.6%	37.8 ± 8.4	
Four times a year	62	12.0%	39.1 ± 10.8	
Once a month	210	40.7%	35.7 ± 9.2	
Once a week	97	18.8%	32.4 ± 8.0	
2+ times/week	52	10.1%	33.7 ± 10.3	
Neighbors	511		0.027	
Never	290	56.8%	37.0 ± 9.9	
Once a year	42	8.2%	36.2 ± 8.9	
Four times a year	47	9.2%	36.3 ± 9.7	
Once a month	91	17.8%	34.6 ± 9.3	
Once a week	30	5.9%	31.1 ± 8.2	
2+ times/week	11	2.2%	35.5 ± 8.4	
Specialists	514			0.020
Never	95	18.5%	39.0 ± 9.6	
Once a year	125	24.3%	36.0 ± 9.7	
Four times a year	134	26.1%	35.2 ± 9.8	
Once a month	142	27.6%	35.0 ± 9.4	
Once a week	9	1.8%	40.0 ± 8.6	
2+ times/week	9	1.8%	33.7 ± 8.7	

*One-way analysis of variance

**Table 6:** Loneliness and channels of information

Channels	Number	Percent	Loneliness Scale score (mean ± SD)	*p* value*
Governmental and corporate homepages	514			0.425
Never	360	70.0%	36.2 ± 9.7	
Once a year	71	13.8%	37.5 ± 10.0	
Four times a year	38	7.4%	35.4 ± 10.1	
Once a month	35	6.8%	33.7 ± 8.0	
Once a week	7	1.4%	32.1 ± 11.2	
2+ times/week	3	0.6%	36.3	
Company and medical homepages	517			0.114
Never	312	60.3%	36.4 ± 9.8	
Once a year	61	11.8%	37.2 ± 10.3	
Four times a year	47	9.1%	35.1 ± 10.4	
Once a month	72	13.9%	35.1 ± 8.6	
Once a week	17	3.3%	31.6 ± 6.9	
2+ times/week	8	1.5%	41.9 ± 7.8	
Personal homepages	512			0.309
Never	288	56.3%	36.6 ± 9.8	
Once a year	41	8.0%	36.9 ± 10.3	
Four times a year	49	9.6%	34.0 ± 8.6	
Once a month	68	13.3%	35.0 ± 10.3	
Once a week	39	7.6%	34.4 ± 8.0	
2+ times/week	27	5.3%	37.4 ± 9.9	
SNS	511			0.013
Never	326	63.8%	37.1 ± 9.6	
Once a year	22	4.3%	37.6 ± 10.3	
Four times a year	22	4.3%	32.9 ± 9.9	
Once a month	59	11.5%	34.3 ± 9.2	
Once a week	46	9.0%	34.0 ± 10.1	
2+ times/week	36	7.0%	32.8 ± 8.8	
Magazines/books	513			0.027
Never	139	27.1%	37.5 ± 10.8	
Once a year	69	13.5%	36.5 ± 9.4	
Four times a year	98	19.1%	37.5 ± 9.5	
Once a month	143	27.9%	34.8 ± 8.7	
Once a week	47	9.2%	34.8 ± 9.7	
2+ times/week	17	3.3%	31.2 ± 8.2	
TV/Radio	513			0.026
Never	197	38.4%	37.4 ± 10.2	
Once a year	58	11.3%	34.9 ± 8.8	
Four times a year	83	16.2%	37.7 ± 9.1	
Once a month	91	17.7%	34.8 ± 9.7	
Once a week	61	11.9%	33.9 ± 8.9	
2+ times/week	23	4.5%	34.1 ± 9.6	
Pamphlets	509			0.133
Never	305	59.9%	36.7 ± 10.1	
Once a year	61	12.0%	34.6 ± 8.8	
Four times a year	62	12.2%	37.7 ± 10.4	
Once a month	65	12.8%	33.6 ± 7.6	
Once a week	13	2.6%	36.4 ± 8.3	
2+ times/week	3	0.6%	37	
Childrearing seminars	513			0.030
Never	292	56.9%	36.3 ± 10.0	
Once a year	88	17.2%	38.1 ± 8.7	
Four times a year	51	9.9%	36.2 ± 10.0	
Once a month	68	13.3%	32.8 ± 8.2	
Once a week	11	2.1%	34.9 ± 10.9	
2+ times/week	3	0.6%	33	

*One-way analysis of variance

SNS Social network site

TV, television

Results from multiple regression analysis are presented in Table 7. Explanatory variables, from the perspective of characteristics, included subjective economic status, health status, IWMS-S (considered an important personal factor), and K6. Age was not selected as an explanatory variable based on previous studies [22, 29] and possible collinearity with other variables. From the perspective of a mother’s relationship with her child, we selected “daycare enrollment” and the four items of LSNS-6 considered to be important social factors (“family,” “friends,” “mom friends,” and “SNS”). Finally, we selected “use frequency of books and magazines” from information sources, and “smartphone use time” from communication devices. Correlation coefficients between explanatory variables were all 0.6 or lower.

**Table 7:** Factors associated with loneliness as determined by multiple regression analysis

	Estimated value	95%CI		*p*-value
Health status				
Very good	Ref.			
Good	1.51	−0.11	3.13	0.067
Fair	3.10	−0.30	6.51	0.074
Subjective economic status				
Stable	Ref.			
Somewhat stable	−3.35	−5.64	−1.05	0.004
Somewhat unstable	−2.47	−4.90	−0.04	0.047
Unstable	0.95	−2.66	4.57	0.605
No daycare	−0.84	−2.26	0.57	0.242
LSNS-6 Family	−0.31	− 0.60	− 0.03	0.032
LSNS-6 Friends	− 0.49	− 0.78	− 0.20	0.001
LSNS-6 Mom friends	0.03	−0.20	0.26	0.812
LSNS-6 SNS	−0.21	−0.38	− 0.03	0.018
Internal Working Model (secure type)	−0.56	− 0.70	−0.41	< 0.001
Magazine and book use	−0.54	−1.59	0.50	0.307
Smartphone use				
< 30 min/day	Ref.			
30 min - 1 h/day	−1.01	−3.63	1.62	0.45
1-2 h/day	−1.72	−4.28	0.84	0.19
2-3 h/day	1.01	−1.70	3.72	0.46
3+ h/day	2.21	−0.83	5.26	0.15
I don’t know	0.33	−5.65	6.31	0.91
Do not own a smartphone	−0.85	−3.87	2.16	0.58
K6 ≥ 5	4.24	2.61	5.86	< 0.001

R-squared value 0.43

R-squared value adjusted for degrees of freedom 0.41

LSNS-6, abbreviated version of the Lubben Social Network Scale

These 11 variables were used as explanatory variables to predict Loneliness Scale scores. Significant associations with high levels of loneliness were found for low scores for the LSNS-6 items of “family,” “friends,” and “SNS”; low IWMS-S scores; and high K6 scores. With regard to subjective economic status, the level of loneliness was significantly higher among people with the highest economic status relative to those with intermediate status. Moreover, those with the lowest economic status had a higher level of loneliness relative to that among those with the highest economic status.

## Discussion

The present study found that low support from SNS friends significantly correlated with high levels of loneliness among mothers raising children, even after adjusting for economic instability, low support from family and friends, low IWMS-S score, and psychological distress. This study is the first to report “personal factors,” “actual use status of communication devices,” and “information sources” as factors associated with loneliness among mothers raising children under the age of 3 years.

We found that loneliness tended to be lower as personal networks created through SNSs as well as traditional networks of family and friends grew. A higher frequency of consultation with parents and friends (more than once/week) was correlated with a higher frequency of SNS use. A previous study reported that Internet communications may reduce feelings of loneliness among the elderly and adolescents [30, 31]. Similarly, the present study showed that SNS use may also reduce loneliness among mothers raising children. The beneficial role of bidirectional information support, including SNSs, provided by public or commercial services warrants further research. Interestingly, loneliness levels among those who used SNSs at a rate of twice or more/week tended to be lower than those of mothers who obtained their information from other sources. The number of friends on SNSs among university students was reported to be associated with social support and health [32]. Thus, the potential of social relationships via SNSs to alleviate loneliness among mothers raising children is worth investigating.

Our results also suggest a U-shaped relationship between the time spent on smartphones and loneliness. The time spent on smartphones by mothers raising children was significant; namely, relative to those who did not have a smartphone, and very little smartphone use (< 0.5 h) was associated with higher levels of loneliness. Longer smartphone use (2–3 h, > 3 h) was also associated with higher levels of loneliness. These results suggest an optimal range of smartphone use which may be associated with lower levels of loneliness. Although determining whether a causal relationship exists will require further investigation, this finding allows us to understand the characteristics of those requiring support, and may provide basic resources and information as we develop ways to support them. Among the few mothers who used tablets/gaming devices, those who spent more time on tablets/gaming devices tended to be lonelier. The length of time on these devices may also reflect the time spent alone in the house, and could indicate that these individuals are moving farther and farther away from social support, e.g., actual human relationships.

A low secure attachment style and psychological distress (K6) were associated with loneliness. The IWMS reveals construction patterns of human relationships, which are strongly correlated with loneliness. Secure attachment represents one’s ability to control negative emotions and behaviors appropriately and achieve a sense of safety through the effective use of assistance received from others. A low secure attachment style increases loneliness because one cannot effectively receive support from others. Ultimately, this is thought to influence one’s responses to changes in actual social relationships, as well as how an individual effectively avoids loneliness by minimizing or lessening it [5]. The fact that loneliness was associated with psychological distress, in the context of mental health of mothers raising children, suggests the need to provide support to these mothers to reduce their loneliness. When providing support to prevent anxiety and child abuse among mothers raising children, in addition to social factors (e.g., SNS use), personal factors such as the tendency to be positively aware of support from others, and the potential for psychological distress should be considered.

With regard to subjective economic status, we found higher levels of loneliness in the lowest economic class, and, unexpectedly, in the highest class. A previous study showed that a low income increased the prevalence of loneliness in an aged population [33]. In addition, Sperlich reported that higher psychosocial stress was found in low income mothers as well as those who were more highly educated [34]. Kahneman argued that above a certain level of stable income, an individual’s emotional well-being is constrained by other factors in their temperament and life circumstances [35]. Our results suggest that mothers derive little psychological benefit from income when economic status is not a daily concern.

Finally, we found that loneliness among teenage mothers raising children was higher than that among mothers older in age. Teenage mothers have characteristically high social and psychological risks [36, 37]. As such, collaboration between medical and public administration personnel becomes increasingly important in the creation of a proactive and continuous social network of support from the time of pregnancy.

### Study limitations and future directions

The present study has several limitations. First, we cannot confirm any causality given the cross-sectional nature of the study. Second, generalizability of the results may be an issue because the questionnaire survey was conducted among mothers who came for regular health check-ups for their children during a specified time period. However, the mean age and employment rate of the participants were similar to those reported in national surveys [38, 39]. Furthermore, the mean Loneliness Scale score for the present study was 36.1; previous domestic studies reported scores of 34–39 [23, 29], suggesting that our participants did not substantially differ from other mothers raising children in Japan. Third, the attendance rate for regular health check-ups for children in Nagahama City is over 90%. Some mothers fail to show up due to illness, a weak household nurturing environment, or work. Women with such backgrounds likely would have higher levels of loneliness due to health and economic issues. As such mothers were not included in the analysis, we may have underestimated the effects of health status and economic stability on loneliness. Fourth, the abbreviated version of the LSNS-6 was originally developed for use with older adult populations. The individual items are not specific to older adults and, since there were no alternatives that targeted young women, we considered it appropriate to use this scale [40–42]. Fifth, we found an association between loneliness and teenage mothers, but our sample size was small. Nonetheless, their loneliness scores were all high (> 40). In addition, the main objective for using various communication devices can vary by use status, but we asked about the time spent on these devices comprehensively as one question, without asking why mothers used their communication devices. Thus, it is unclear whether loneliness was affected specifically by tablet use or gaming devices, or by SNS use or talking. Further studies will be needed regarding how communication device use may impact the loneliness of mothers raising children.

Despite these limitations, our study is the first to demonstrate the importance of considering social networks, including communication device and SNS use, as potential ways to target loneliness among mothers raising children.

## Conclusions

Loneliness among mothers raising children was found to be associated with sparse social relationships with others including family, friends, and SNSs; low sense of “secure” in the Internal Working Model Scale; and a high likelihood for psychological distress. Our findings may serve as a basic resource when considering how to provide the appropriate support structure to mothers raising children.

## Additional files

Additional file 1: Questionnaire. The study questionnaire translated into English. The questionnaire contained 71 items with the following content: basic characteristics, attachment patterns (patterns showing the tendency towards easy acceptance of help from others), loneliness, psychological distress, social networks (number of associated people and support), and types of communication devices and information sources. (DOCX 40 kb)

Additional file 2: Questionnaire (Japanese). The study questionnaire in Japanese. (DOCX 57 kb)

Additional file 3: Time of smartphone use and frequency of SNS. (DOCX 18 kb)

Additional file 4: Consultation frequency of parents, friends, and SNS. (DOCX 18 kb)

Additional file 5: “Secure” subscale scores of the Internal Working Model Scale (Mean, Standard division) and frequency of various consultations and channels. (DOCX 21 kb)

## Abbreviations

ANOVA: Analysis of variance
IWMS: Internal Working Model Scale
IWMS-S: “Secure” subscale of Internal Working Model Scale
LSNS: Lubben Social Network Scale
LSNS-6: Abbreviated version of the Lubben Social Network Scale
SD: Standard deviation
SNS(s): Social Network Site(s)
TV: Television
