# Supplementary material for: Loneliness among mothers raising children under the age of 3 years and predictors with special reference to the use of SNS: a community-based cross-sectional study
Source: BMC Womens Health. 2018 Aug 16;18:131. doi: 10.1186/s12905-018-0625-x (PMC6094879; doi:10.1186/s12905-018-0625-x)
Supplement: Supplementary file 1 — Questionnaire. The study questionnaire translated into English. The questionnaire contained 71 items with the following content: basic characteristics, attachment patterns (patterns showing the tendency towards easy acceptance of help from others), loneliness, psychological distress, social networks (number of associated people and support), and types of communication devices and information sources. (DOCX 40 kb) [file 12905_2018_625_MOESM1_ESM.docx]

Questionnaire

**[1].** Please consider the following questions, and circle the applicable answer.

| 1－①How old are you?　　　　　　　　　　　　　　　　　　　　years old |
| --- |
| 1－②Below we will ask you about your children, including the number of children and their gender and age. If you have more than one child, then please answer the question below for all children in age order. |
| ・ year month M/F Child’s health condition (1.Very good 2.Good 3.Fair 4.Poor) |
| ・ year month M/F Child’s health condition (1.Very good 2.Good 3.Fair 4.Poor) |
| ・ year month M/F Child’s health condition (1.Very good 2.Good 3.Fair 4.Poor) |
| ・ year month M/F Child’s health condition (1.Very good 2.Good 3.Fair 4.Poor) |
| ・ year month M/F Child’s health condition (1.Very good 2.Good 3.Fair 4.Poor) |
| 2－①Are you working?　　　　　　　　　　　　　　　　　　　　　　 1.　Yes　　　　　　　2.　No |
| 2－②Does your child(ren) go to a childcare facility, nursery, or kindergarten?  　 1.　Yes 　　　　2.　No |
| 2－②What is your current health status?  1．Very good　　　　　　　　　　2．Good　　　　　　　　　3．Fair　　　　 4．Poor |
| 2－③How do you feel the finance situation of your family?  1．Stable 　　　　　2．Somewhat stable 　　　　　3．Somewhat unstable　　　　　4．Unstable |
| 3－①Are you married?　　　　1．Single　　 　2．Married　　 3．Lost　　　 　4．Divorced |
| ② Please answer the questions below if you are married.  Does your spouse cooperate with child rearing or housekeeping?　　　　 1.Yes　　　　　　　　　　　2.No |
| 3－③Do you live with your or your spouse’s parents? 　　　　 1.Yes　　　　　　　　　　2.No |
| 3－④What is the highest education you have completed?  1．Junior high school　　　2．High school　　3．Trade school/junior college　　4．University/graduate school |

4．If you have inquired about child rearing or health, please fill in the blank below (e.g., breast milk, vaccination, allergy, fever).

**
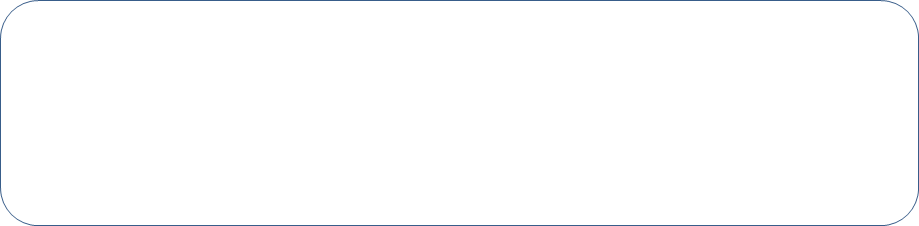
**

5. In the past three months, how often did you use the communication devices below on average per day? If it varies by day, please circle the most applicable number for average daily usage among 0 to 6 (e.g., if you use a smartphone for an average of 2.5 hours a day, please circle 4).

|  | Don’t have | <0.5 hours | 0.5-1 hours | 1-2 hours | 2-3 hours | ≧3 hours | Don’t know |
| --- | --- | --- | --- | --- | --- | --- | --- |
| e.g.  2．Smartphones | 0 | 1 | 2 | 3 |  | 5 | 6 |
| 1．Traditional cell phones | 0 | 1 | 2 | 3 | 4 | 5 | 6 |
| 2．Smartphones | 0 | 1 | 2 | 3 | 4 | 5 | 6 |
| 3．Tablets | 0 | 1 | 2 | 3 | 4 | 5 | 6 |
| 4．Personal computers | 0 | 1 | 2 | 3 | 4 | 5 | 6 |

**[2].** Since the birth of your child, how often have you used the following information sources to look for childcare and health information? Please circle the applicable number.

|  | Never | Once a year | Four times a year | Once a month | Once a week | 2+times/week |
| --- | --- | --- | --- | --- | --- | --- |
| 1．Parents | 0 | 1 | 2 | 3 | 4 | 5 |
| 2．Friends | 0 | 1 | 2 | 3 | 4 | 5 |
| 3．Neighbors | 0 | 1 | 2 | 3 | 4 | 5 |
| 4．Specialists(Doctor / midwife master /  Nurse, pharmacist, public health nurse, nursery teacher etc) | 0 | 1 | 2 | 3 | 4 | 5 |
| 5．Governmental and corporate homepages | 0 | 1 | 2 | 3 | 4 | 5 |
| 6．Company and medical homepages | 0 | 1 | 2 | 3 | 4 | 5 |
| 7．Personal homepages | 0 | 1 | 2 | 3 | 4 | 5 |
| 8． SNS (LINE・Twitter・Facebook・mixi etc) | 0 | 1 | 2 | 3 | 4 | 5 |
| 9．　Magazines/Books | 0 | 1 | 2 | 3 | 4 | 5 |
| 10． TV/Radio | 0 | 1 | 2 | 3 | 4 | 5 |
| 11． Pamphlets | 0 | 1 | 2 | 3 | 4 | 5 |
| 12．Childrearing seminars | 0 | 1 | 2 | 3 | 4 | 5 |

**[3]**. Indicate how often you feel the way described in each of the following statements. Circle one number for each.

|  | Never | Rarely | Sometimes | Often |
| --- | --- | --- | --- | --- |
| 1．I feel in tune with the people around me | 1 | 2 | 3 | 4 |
| 2．I lack companionship | 1 | 2 | 3 | 4 |
| 3．There is no one I can turn to | 1 | 2 | 3 | 4 |
| 4．I do not feel alone | 1 | 2 | 3 | 4 |
| 5．I feel part of a group of friends | 1 | 2 | 3 | 4 |
| 6．I have a lot in common with the people around me | 1 | 2 | 3 | 4 |
| 7．I am no longer close to anyone | 1 | 2 | 3 | 4 |
| 8．My interests and ideas are not shared by those around me | 1 | 2 | 3 | 4 |
| 9．I am an outgoing person | 1 | 2 | 3 | 4 |
| 10．There are people I feel close to | 1 | 2 | 3 | 4 |
| 11．I feel left out | 1 | 2 | 3 | 4 |
| 12．My social relationships are superficial | 1 | 2 | 3 | 4 |
| 13．No one really knows me well | 1 | 2 | 3 | 4 |
| 14．I feel isolated from others | 1 | 2 | 3 | 4 |
| 15．I can find companionship when I want it | 1 | 2 | 3 | 4 |
| 16．There are people who really understand me | 1 | 2 | 3 | 4 |
| 17．I am unhappy being so withdrawn | 1 | 2 | 3 | 4 |
| 18．People are around me but not with me | 1 | 2 | 3 | 4 |
| 19．there are people I can talk to | 1 | 2 | 3 | 4 |
| 20．There are people I can turn to | 1 | 2 | 3 | 4 |

**[4]**. We will ask questions about your family and friends. Please circle the applicable number.

| FAMILY: Considering the people to whom you are related either by birth or marriage… | None | One | Two | Three or four | Five thru eight | Nine or more |
| --- | --- | --- | --- | --- | --- | --- |
| 1.How many relatives do you see or hear from at least once a month? | 0 | 1 | 2 | 3 | 4 | 5 |
| 2．How many relatives do you feel close to such that you could call on them for help? | 0 | 1 | 2 | 3 | 4 | 5 |
| 3．How many relatives do you feel at ease with that you can talk about private matters? | 0 | 1 | 2 | 3 | 4 | 5 |

| FRIENDSHIPS: Considering all of your friends including those who live in your neighborhood… | None | One | Two | Three or four | Five thru eight | Nine or more |
| --- | --- | --- | --- | --- | --- | --- |
| 1.How many of your friends do you see or hear from at least once a month? | 0 | 1 | 2 | 3 | 4 | 5 |
| 2．How many friends do you feel close to such that you could call on them for help? | 0 | 1 | 2 | 3 | 4 | 5 |
| 3．How many friends do you feel at ease with that you can talk about private matters? | 0 | 1 | 2 | 3 | 4 | 5 |

| MOM FRIENDSHIPS: Considering all of your Mom friends who are mothers acquainted through one’s children… | None | One | Two | Three or four | Five thru eight | Nine or more |
| --- | --- | --- | --- | --- | --- | --- |
| 1.How many of your Mom friends do you see or hear from at least once a month? | 0 | 1 | 2 | 3 | 4 | 5 |
| 2．How many Mom friends do you feel close to such that you could call on them for help? | 0 | 1 | 2 | 3 | 4 | 5 |
| 3．How many Mom friends do you feel at ease with that you can talk about private matters? | 0 | 1 | 2 | 3 | 4 | 5 |

| SNS FRIENDSHIPS: Considering all of your friends who are interacting by LINE, Facebook or Twitter… | None | One | Two | Three or four | Five thru eight | Nine or more |
| --- | --- | --- | --- | --- | --- | --- |
| 1. How many of your SNS friends do you see or hear from at least once a month? | 0 | 1 | 2 | 3 | 4 | 5 |
| 2．How many SNS friends do you feel close to such that you could call on them for help? | 0 | 1 | 2 | 3 | 4 | 5 |
| 3．How many SNS friends do you feel at ease with that you can talk about private matters? | 0 | 1 | 2 | 3 | 4 | 5 |

**[5]**. The following questions ask about how you have been feeling during the past 30 days. For each question, please circle the number that best describes how often you had this feeling.

|  | None of the time | A little of the time | Some of the time | Most of the time | All of the time |
| --- | --- | --- | --- | --- | --- |
| 1．…nervous? | 0 | 1 | 2 | 3 | 4 |
| 2．…hopeless? | 0 | 1 | 2 | 3 | 4 |
| 3．…restless or fidgety? | 0 | 1 | 2 | 3 | 4 |
| 4．…so depressed that nothing could cheer you up? | 0 | 1 | 2 | 3 | 4 |
| 5．…that everything was an effort? | 0 | 1 | 2 | 3 | 4 |
| 6．…worthless? | 0 | 1 | 2 | 3 | 4 |

**[6]**. To what extent does the content of each of the items below describe you normally? Please circle the applicable number.

|  | Strongly disagree | Disagree | Slightly disagree | Slightly agree | Agree | Strongly agree |
| --- | --- | --- | --- | --- | --- | --- |
| 1．I am easier to get to know than most people. | 1 | 2 | 3 | 4 | 5 | 6 |
| 2．I find it relatively easy to get close to others. | 1 | 2 | 3 | 4 | 5 | 6 |
| 3．I think I am a likable person. | 1 | 2 | 3 | 4 | 5 | 6 |
| 4．People almost always like me. | 1 | 2 | 3 | 4 | 5 | 6 |
| 5．I am comfortable depending on others and having them depend on me. | 1 | 2 | 3 | 4 | 5 | 6 |
| 6．I feel confident that I can get along well with a stranger. | 1 | 2 | 3 | 4 | 5 | 6 |

Thank you for your cooperation.
