# Supplementary material for: Loneliness among mothers raising children under the age of 3 years and predictors with special reference to the use of SNS: a community-based cross-sectional study
Source: BMC Womens Health. 2018 Aug 16;18:131. doi: 10.1186/s12905-018-0625-x (PMC6094879; doi:10.1186/s12905-018-0625-x)
Supplement: Supplementary file 2 — Questionnaire (Japanese). The study questionnaire in Japanese. (DOCX 57 kb) [file 12905_2018_625_MOESM2_ESM.docx]

**Questionnaire (Japanese)**

**アンケート**

**Ⅰ．はじめに、ご自身のことをおききします。番号がある場合は、当てはまる番号に○をつけてください。**

| 1－①あなたの年齢　　　　　　　　　　　　　　　　　　　　　歳 |
| --- |
| 1－②お子さんの人数と月齢、性別をお聞きします。2人以上いらっしゃるときは、上のお子さんから  お書きください。 |
| ・　　　歳　　か月　　男・女　　お子さんの健康状態：（1．良い　2．まあ良い　3．あまりよくない　4．よくない） |
| ・　　　歳　　か月　　男・女　　お子さんの健康状態：（　1．良い　2．まあ良い　3．あまりよくない　4．よくない） |
| ・　　　歳　　か月　　男・女　　お子さんの健康状態：（　1．良い　2．まあ良い　3．あまりよくない　4．よくない） |
| ・　　　歳　　か月　　男・女　　お子さんの健康状態：（　1．良い　2．まあ良い　3．あまりよくない　4．よくない） |
| ・　　　歳　　か月　　男・女　　お子さんの健康状態：（　1．良い　2．まあ良い　3．あまりよくない　4．よくない） |
| 2－①仕事をもっていますか　　　　　　　　　　　　　　　　　　　　　　 1.　はい　　　　　　　2.　いいえ |
| 2－②保育園や幼稚園などに預けているお子さんはいますか　　 1.　はい　　　　　　　2.　いいえ |
| 2－②ご自身の健康状態は  1．よい　　　　　　　　　　2．まあよい　　　　　　　　　3．あまりよくない　　　　4．よくない |
| 2－③ご家庭の経済状況をおききします。  1．ゆとりがある　　　　　2．少しゆとりがある　　　　　3．少し苦しい　　　　　4．苦しい |
| 3－①ご結婚されていますか　　　　1．未婚　　 　2．既婚（事実婚を含む）　　 3．死別　　　 　4．離別 |
| ②、①で2.　既婚　と答えた方におききします。  配偶者は育児や家事に協力的ですか　　　　　　　　　　　　　　　1.はい　　　　　　　　　　　2.いいえ |
| 3－③同居する両親（義父母含む）はいらっしゃいますか　　　　1.あり　　　　　　　　　　　2.なし |
| 3－④最後に卒業された学校をお答えください  1．中学校　　　　2．高等学校　　3．専門学校・短大　　4．大学・大学院 |

４．育児やからだのことなどで、人に聞いたり調べたりしたことがあれば、例を参考に具体的に下の空欄にお書きください。

例：母乳のこと　予防接種のこと　アレルギーのこと　発熱のことなど

**
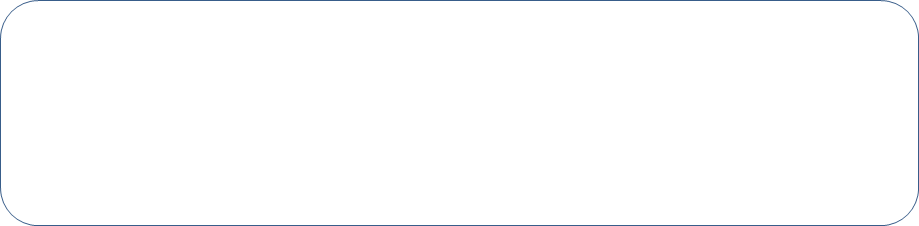
**

**５．携帯などの通信機器の使用の状況をおききします。**

**次の通信機器を、この3か月の間で、1日に平均どれくらい使用しましたか。日によって異なる場合は、最も一般的だと思われる日を想定して御回答ください。以下の0から6までの選択肢の中で、一日の平均の使用時間の最も当てはまる番号欄に〇をつけてください。**

**例**

**スマートフォンを1日に平均２時間３０分程度使われる場合は、4に〇を記入してください。**

|  | 持っていない | 30分未満 | 30分～1時間未満 | 1～2時間  未満 | 2～3時間  未満 | 3時間以上 | わからない |
| --- | --- | --- | --- | --- | --- | --- | --- |
| 例  2．スマートフォン | 0 | 1 | 2 | 3 |  | 5 | 6 |
| 1．従来の携帯電話 | 0 | 1 | 2 | 3 | 4 | 5 | 6 |
| 2．スマートフォン | 0 | 1 | 2 | 3 | 4 | 5 | 6 |
| 3．タブレット端末  （ゲーム機を含む） | 0 | 1 | 2 | 3 | 4 | 5 | 6 |
| 4．パソコン | 0 | 1 | 2 | 3 | 4 | 5 | 6 |

**Ⅱあなたは、育児やからだのことでわからないとき、お子さんが生まれてから今までに**

**どのような情報源をどれぐらいの頻度で使いましたか。**

**それぞれの情報源について、0から5までであてはまる番号に○をつけて下さい。**

|  | 利用しなかった | 年に1回程度 | 年に4回程度 | 月に1回程度 | 週に1回程度 | 週に2回以上 |
| --- | --- | --- | --- | --- | --- | --- |
| 1．親 | 0 | 1 | 2 | 3 | 4 | 5 |
| 2．友人 | 0 | 1 | 2 | 3 | 4 | 5 |
| 3．近所に住む人 | 0 | 1 | 2 | 3 | 4 | 5 |
| 4．専門家や医療従事者（医師・助産師・  看護師・薬剤師・保健師・保育士等） | 0 | 1 | 2 | 3 | 4 | 5 |
| 5．地方自治体・国の公共機関のホームページ | 0 | 1 | 2 | 3 | 4 | 5 |
| 6．企業や医療機関のホームページ | 0 | 1 | 2 | 3 | 4 | 5 |
| 7．個人のホームページ・ブログ | 0 | 1 | 2 | 3 | 4 | 5 |
| 8． SNS（・・・  など） | 0 | 1 | 2 | 3 | 4 | 5 |
| 9．　雑誌・書籍 | 0 | 1 | 2 | 3 | 4 | 5 |
| 10． テレビ・ラジオ・新聞 | 0 | 1 | 2 | 3 | 4 | 5 |
| 11． パンフレット | 0 | 1 | 2 | 3 | 4 | 5 |
| 12．育児セミナー、母親教室 | 0 | 1 | 2 | 3 | 4 | 5 |

**Ⅲ．以下の各項目の内容は、普段のあなたにどの程度あてはまりますか。**

**あてはまる番号に○をつけてください。**

|  | 決して  感じない | めったに  感じない | 時々感じる | しばしば  感じる |
| --- | --- | --- | --- | --- |
| 1．私は自分の周囲の人たちと調子よくいっている | 1 | 2 | 3 | 4 |
| 2．私は人とのつきあいがない | 1 | 2 | 3 | 4 |
| 3．私には頼りにできるひとがだれもいない | 1 | 2 | 3 | 4 |
| 4．私はひとりぼっちではない | 1 | 2 | 3 | 4 |
| 5．私は親しい友だちの気心がわかる | 1 | 2 | 3 | 4 |
| 6．私は自分の周囲の人たちと共通点が多い | 1 | 2 | 3 | 4 |
| 7．私は今、誰とでも親しくしていない | 1 | 2 | 3 | 4 |
| 8．私の興味や考えは、私の周囲の人たちとは  ちがう | 1 | 2 | 3 | 4 |
| 9．私は外出好きの人間である | 1 | 2 | 3 | 4 |
| 10．私には親密感の持てる人たちがいる | 1 | 2 | 3 | 4 |
| 11．私は疎外されている | 1 | 2 | 3 | 4 |
|  | 決して  感じない | めったに  感じない | 時々感じる | しばしば  感じる |
| 12．私の社会的なつながりはうわべだけのものである | 1 | 2 | 3 | 4 |
| 13．私をよく知っている人は誰もいない | 1 | 2 | 3 | 4 |
| 14．私は他の人たちから孤立している | 1 | 2 | 3 | 4 |
| 15．私はその気になれば、人と付き合うことが  できる | 1 | 2 | 3 | 4 |
| 16．私を本当に理解している人たちがいる | 1 | 2 | 3 | 4 |
| 17．私は本当に引っ込み思案なのでみじめである | 1 | 2 | 3 | 4 |
| 18．私には知人がいるが、気心の知れた人は  いない | 1 | 2 | 3 | 4 |
| 19．私には話し合える人たちがいる | 1 | 2 | 3 | 4 |
| 20．私には頼れる人たちがいる | 1 | 2 | 3 | 4 |

**Ⅳ．家族や友人についておききします。当てはまる番号に〇をつけてください。**

| 家族、ここでは家族や親せきなどについて考えます | いない | 1人 | 2人 | 3~4人 | 5~8人 | 9人以上 |
| --- | --- | --- | --- | --- | --- | --- |
| 1.少なくとも月に1回、会ったり話をしたりする家族や親せきは何人いますか？ | 0 | 1 | 2 | 3 | 4 | 5 |
| 2．あなたが、個人的なことでも話すことができるくらい気楽に感じられる家族や親せきは何人いますか？ | 0 | 1 | 2 | 3 | 4 | 5 |
| 3．あなたが、助けを求めることができるくらい親しく感じられる家族や親せきは何人いますか？ | 0 | 1 | 2 | 3 | 4 | 5 |

| 友人関係、ここでは近くに住んでいる人を含むあなたの友人全体について考えます | いない | 1人 | 2人 | 3~4人 | 5~8人 | 9人以上 |
| --- | --- | --- | --- | --- | --- | --- |
| 1.少なくとも月に1回、会ったり話をしたりする友人は何人いますか？ | 0 | 1 | 2 | 3 | 4 | 5 |
| 2．あなたが、個人的なことでも話すことができるくらい気楽に感じられる友人は何人いますか？ | 0 | 1 | 2 | 3 | 4 | 5 |
| 3．あなたが、助けを求めることができるくらい親しく感じられる友人は何人いますか？ | 0 | 1 | 2 | 3 | 4 | 5 |

| ママ友達、ここでは妊娠・出産・育児を通じて知り合った友人について考えます | いない | 1人 | 2人 | 3~4人 | 5~8人 | 9人以上 |
| --- | --- | --- | --- | --- | --- | --- |
| 1.少なくとも月に1回、会ったり話をしたりするママ友達は何人いますか？ | 0 | 1 | 2 | 3 | 4 | 5 |
| 2．あなたが、個人的なことでも話すことができるくらい気楽に感じられるママ友達は何人いますか？ | 0 | 1 | 2 | 3 | 4 | 5 |
| 3．あなたが、助けを求めることができるくらい親しく感じられるママ友達は何人いますか？ | 0 | 1 | 2 | 3 | 4 | 5 |

| SNSの友人、ここではLINEやFacebookやTwitterでやりとりのある友人について考えます | いない | 1人 | 2人 | 3~4人 | 5~8人 | 9人以上 |
| --- | --- | --- | --- | --- | --- | --- |
| 1.少なくとも月に1回、やりとりをするSNSの友人は何人いますか？ | 0 | 1 | 2 | 3 | 4 | 5 |
| 2．あなたが、個人的なことでも話すことができるくらい気楽に感じられるSNSの友人は何人いますか？ | 0 | 1 | 2 | 3 | 4 | 5 |
| 3．あなたが、助けを求めることができるくらい親しく感じられるSNSの友人は何人いますか？ | 0 | 1 | 2 | 3 | 4 | 5 |

**Ⅴ．過去30日の間にどれくらいの頻度で次のことがありましたか。あてはまる番号に○をつけてください。**

|  | 全くない | 少しだけ | ときどき | たいてい | いつも |
| --- | --- | --- | --- | --- | --- |
| 1．神経過敏に感じましたか | 0 | 1 | 2 | 3 | 4 |
| 2．絶望的だと感じましたか | 0 | 1 | 2 | 3 | 4 |
| 3．そわそわ、落ち着かなく感じましたか | 0 | 1 | 2 | 3 | 4 |
| 4．気分が沈み込んで、何が起こっても気が晴れないように感じましたか | 0 | 1 | 2 | 3 | 4 |
| 5．何をするのも骨折りだと感じましたか | 0 | 1 | 2 | 3 | 4 |
| 6．自分は価値のない人間だと感じましたか | 0 | 1 | 2 | 3 | 4 |

**Ⅵ．以下の各項目の内容は、普段のあなたにどの程度あてはまりますか。番号に○をつけてください。**

|  | 全く当てはまらない | あてはまらない | あまり当てはまらない | やや  当てはまる | 当てはまる | 非常によく  当てはまる |
| --- | --- | --- | --- | --- | --- | --- |
| 1．私は知り合いができやすい方だ。 | 1 | 2 | 3 | 4 | 5 | 6 |
| 2．私はすぐに人と親しくなる方だ。 | 1 | 2 | 3 | 4 | 5 | 6 |
| 3．私は人に好かれやすい性質だと思う。 | 1 | 2 | 3 | 4 | 5 | 6 |
| 4．たいていの人は私のことを好いてくれていると思う。 | 1 | 2 | 3 | 4 | 5 | 6 |
| 5．気軽に頼ったり頼られたりすることができる。 | 1 | 2 | 3 | 4 | 5 | 6 |
| 6．初めて会った人とでもうまくやっていける自信がある。 | 1 | 2 | 3 | 4 | 5 | 6 |

ご協力ありがとうございました。
