# Supplementary material for: Loneliness among mothers raising children under the age of 3 years and predictors with special reference to the use of SNS: a community-based cross-sectional study
Source: BMC Womens Health. 2018 Aug 16;18:131. doi: 10.1186/s12905-018-0625-x (PMC6094879; doi:10.1186/s12905-018-0625-x)
Supplement: Supplementary file 3 — Time of smartphone use and frequency of SNS. (DOCX 18 kb) [file 12905_2018_625_MOESM3_ESM.docx]

**Additional file 3**

**eTable 1. Time of smartphone use and frequency of SNS**

|  |  | Frequency of SNS | | | | | | | |  |
| --- | --- | --- | --- | --- | --- | --- | --- | --- | --- | --- |
|  |  | 0 | |  | <1/week | |  | ≧1/week | |  |
| Smartphone use |  | n | % |  | n | % |  | n | % | p value* |
| Don't have |  | 59 | 92.2 |  | 3 | 4.7 |  | 2 | 3.1 | <0.001 |
| <0.5 hours |  | 35 | 72.9 |  | 11 | 22.9 |  | 2 | 4.2 |  |
| 0.5-1 hour |  | 66 | 60.0 |  | 25 | 22.7 |  | 19 | 17.3 |  |
| 1-2 hours |  | 75 | 55.6 |  | 31 | 23.0 |  | 29 | 21.5 |  |
| 2-3 hours |  | 54 | 57.5 |  | 21 | 22.3 |  | 19 | 20.2 |  |
| ≧3 hours |  | 30 | 58.8 |  | 12 | 23.5 |  | 9 | 17.7 |  |
| No answer |  | 6 | 75.0 |  | 0 | 0.0 |  | 2 | 25.0 |  |
| *χ^２^ test |  |  |  |  |  |  |  |  |  |  |
| SNS Social Network Site | | | | | | | | | | |
