# Supplementary material for: Loneliness among mothers raising children under the age of 3 years and predictors with special reference to the use of SNS: a community-based cross-sectional study
Source: BMC Womens Health. 2018 Aug 16;18:131. doi: 10.1186/s12905-018-0625-x (PMC6094879; doi:10.1186/s12905-018-0625-x)
Supplement: Supplementary file 4 — Consultation frequency of parents, friends, and SNS. (DOCX 18 kb) [file 12905_2018_625_MOESM4_ESM.docx]

**Additional file 4**

**eTable 2. Consultation frequency of parents, friends and SNS**

|  |  |  | Frequency of SNS | | | | | | | | |
| --- | --- | --- | --- | --- | --- | --- | --- | --- | --- | --- | --- |
|  |  |  | 0 | |  | <1/week | |  | ≧1/week | |  |
|  |  |  | n | % |  | n | % |  | n | % | p value* |
| Frequency of parents | <1/week |  | 150 | 67.9 |  | 46 | 20.8 |  | 25 | 11.3 | 0.039 |
|  | ≧1/week |  | 173 | 60.7 |  | 56 | 19.6 |  | 56 | 19.6 |  |
|  |  |  |  |  |  |  |  |  |  |  |  |
| Frequency of friends | <1/week |  | 253 | 70.5 |  | 72 | 20.1 |  | 34 | 9.5 | <0.001 |
|  | ≧1/week |  | 70 | 47.3 |  | 31 | 20.9 |  | 47 | 31.8 |  |
| *χ^２^ test |  |  |  |  |  |  |  |  |  |  |  |
| SNS Social Network Site | | |  |  |  |  |  |  |  |  |  |
