# Supplementary material for: Loneliness among mothers raising children under the age of 3 years and predictors with special reference to the use of SNS: a community-based cross-sectional study
Source: BMC Womens Health. 2018 Aug 16;18:131. doi: 10.1186/s12905-018-0625-x (PMC6094879; doi:10.1186/s12905-018-0625-x)
Supplement: Supplementary file 5 — “Secure” subscale scores of the Internal Working Model Scale (Mean, Standard division) and frequency of various consultations and channels. (DOCX 21 kb) [file 12905_2018_625_MOESM5_ESM.docx]

**Additional file 5**

**eTable 3. “Secure” subscale scores of the Internal Working Model Scale (Mean, Standard division) and frequency of various consultations and channels**

|  | Frequency | | | | | | | | | | | | | | | | | | |
| --- | --- | --- | --- | --- | --- | --- | --- | --- | --- | --- | --- | --- | --- | --- | --- | --- | --- | --- | --- |
| Consultations/channels | 0 | | 1/year | | 4/year | | | 1/month | | | | 1/week | | | | 2/week | | | p-value* |
| Parents | 21.1 | 7.6 | 21.7 | 6.2 | 20.8 | 5.0 | | 19.9 | 5.2 | | | 20.5 | | 5.4 | | 21.2 | 5.4 | | 0.432 |
| Friends | 18.9 | 6.3 | 19.7 | 4.3 | 18.6 | 5.7 | | 20.6 | 5.1 | | | 21.9 | | 5.0 | | 23.1 | 6.0 | | <0.001 |
| Neighbors | 20.0 | 5.8 | 19.7 | 4.9 | 20.5 | 4.8 | | 22.0 | 5.1 | | | 23.1 | | 4.0 | | 22.1 | 3.4 | | 0.003 |
| Specialists | 19.8 | 6.0 | 20.2 | 5.1 | 20.9 | 5.5 | | 21.0 | 5.2 | | | 20.5 | | 4.3 | | 23.0 | 3.6 | | 0.338 |
| Governmental and corporate homepages | 20.4 | 5.4 | 20.7 | 5.7 | 22.1 | 5.2 | | 20.7 | 5.2 | | | 20.4 | | 5.1 | | 27.0 | 4.2 | | 0.308 |
| Company and medical homepages | 20.4 | 5.6 | 20.8 | 5.5 | 20.5 | 4.6 | | 21.3 | 4.9 | | | 21.2 | | 5.8 | | 18.3 | 7.7 | | 0.710 |
| Personal homepages | 20.3 | 5.6 | 20.8 | 6.1 | 20.6 | 5.5 | | 21.4 | 4.5 | | | 21.5 | | 4.4 | | 20.9 | 5.8 | | 0.586 |
| SNS | 19.9 | 5.4 | 20.4 | 5.4 | 20.5 | 5.1 | | 21.3 | 5.4 | | | 22.9 | | 5.7 | | 23.6 | 4.8 | | <0.001 |
| Magazines/books | 19.7 | 6.6 | 20.2 | 5.6 | 19.9 | 5.0 | | 21.5 | 4.9 | | | 21.7 | | 3.7 | | 21.9 | 4.5 | | 0.033 |
| TV/Radio | 20.0 | 6.2 | 19.9 | 4.8 | 20.1 | 4.2 | | 21.8 | 4.8 | | | 21.8 | | 5.5 | | 21.4 | 5.6 | | 0.040 |
| Pamphlets | 20.2 | 5.8 | 20.6 | 5.9 | 21.3 | 4.5 | | 21.7 | 4.4 | | | 21.5 | | 5.1 | | 19.0 | n.a. | | 0.345 |
| Childrearing seminars | 20.4 | 5.6 | 19.4 | 5.1 | 20.8 | 5.0 | | 22.6 | 4.7 | | | 21.0 | | 6.7 | | 26.0 | 4.2 | | 0.007 |
| * One-way analysis of variance | | | | | | | | | | | | | | | | | | | |
| SNS Social Network Site | | | | | | |  | | |  |  | |  | |  | | |  |  |
| TV Television | | | | | | | | | | | | |  | |  | | |  |  |
